# Systematic evaluation of the degraded products evolved from the hydrothermal pretreatment of sweet sorghum stems

**DOI:** 10.1186/s13068-015-0223-1

**Published:** 2015-03-04

**Authors:** Shaolong Sun, Jialong Wen, Shaoni Sun, Run-Cang Sun

**Affiliations:** No. 35 Tsing Hua East Road, Haidian District, Beijing Key Laboratory of Lignocellulosic Chemistry, Beijing Forestry University, Beijing, 100083 China

**Keywords:** Sweet sorghum stem, Hydrothermal pretreatment, Degraded products, Heteronuclear single quantum coherence (HSQC)

## Abstract

**Background:**

Conversion of plant cell walls to bioethanol and bio-based chemicals requires pretreatment as a necessary step to reduce recalcitrance of cell walls to enzymatic and microbial deconstruction. In this study, the sweet sorghum stems were subjected to various hydrothermal pretreatment processes (110°C to 230°C, 0.5 to 2.0 h), and the focus of this work is to systematically evaluate the degraded products of polysaccharides and lignins in the liquor phase obtained during the pretreatment process.

**Results:**

The maximum yield of xylooligosaccharides (52.25%) with a relatively low level of xylose and other degraded products was achieved at a relatively high pretreatment temperature (170°C) for a short reaction time (0.5 h). Higher temperature (>170°C) and/or longer reaction time (>0.5 h at 170°C) resulted in a decreasing yield of xylooligosaccharides, but increased the concentration of arabinose and galactose. The xylooligosaccharides obtained are composed of xylopyranosyl residues, together with lower amounts of 4-*O*-Me-*α*-D-GlcpA units. Meanwhile, the concentrations of the degraded products (especially furfural) increased as a function of pretreatment temperature and time. Molecular weights of the water-soluble polysaccharides and lignins indicated that the degradation of the polysaccharides and lignins occurred during the conditions of harsh hydrothermal pretreatment. In addition, the water-soluble polysaccharides (rich in xylan) and water-soluble lignins (rich in *β*-*O*-4 linkages) were obtained at 170°C for 1.0 h.

**Conclusions:**

The present study demonstrated that the hydrothermal pretreatment condition had a remarkable impact on the compositions and the chemical structures of the degraded products. An extensive understanding of the degraded products from polysaccharides and lignins during the hydrothermal pretreatment will be beneficial to value-added applications of multiple chemicals in the biorefinery for bioethanol industry.

## Background

Lignocelluloses have huge growth potential due to their contribution to the bioethanol production and to decrease CO_2_ emission and global warming in recent years [1,2]. Sweet sorghum is considered as one of the potential renewable sources of energy for economic development and environmental sustainability, owing to its wide adaptability and high concentration of soluble sugars [3]. This plant could be effectively used as a source of fodder (stem), food (grains), and also as feedstock (cellulose, hemicelluloses, and lignins) for the production of industrial chemicals [3,4]. However, in the production of bioethanol, the direct enzymatic hydrolysis of cellulose is greatly hampered by the dense and complex architectural structure of biomass [5]. Since pretreatment accelerates enzymes accessing the rigid structure of biomass for maximum yield of fermentable sugar, pretreatment is considered as a crucial link prior to biomass enzymatic hydrolysis [5,6]. By now, a variety of physical, chemical, mechanical, and biological pretreatment techniques have been developed, including the developments of steam explosion, organosolv pretreatment, and hydrothermal pretreatment (HTP) [7-9]. Among them, HTP is a handy and eco-friendly technology for the pretreatment of lignocelluloses as compared to others, since the medium only contains water, avoiding corrosion of equipment [10]. In addition, HTP can result in structural changes of lignins and cellulose as well as solubilization of hemicelluloses, which in turn contribute to the improved enzyme accessibility in pretreated biomass [10]. Furthermore, HTP can also produce some important by-products, such as xylo-oligosaccharides (XOS), and some other chemicals (for example, furfural, formic acid, and acetic acid) [11]. The fundamental understanding of the degraded products can increase high-value utilization of biomass in a biorefinery process [12]. Therefore, it is necessary to characterize the degraded products in the liquors recovered during the HTP, which will enhance biorefinery viability in the future.

**Figure 1:**
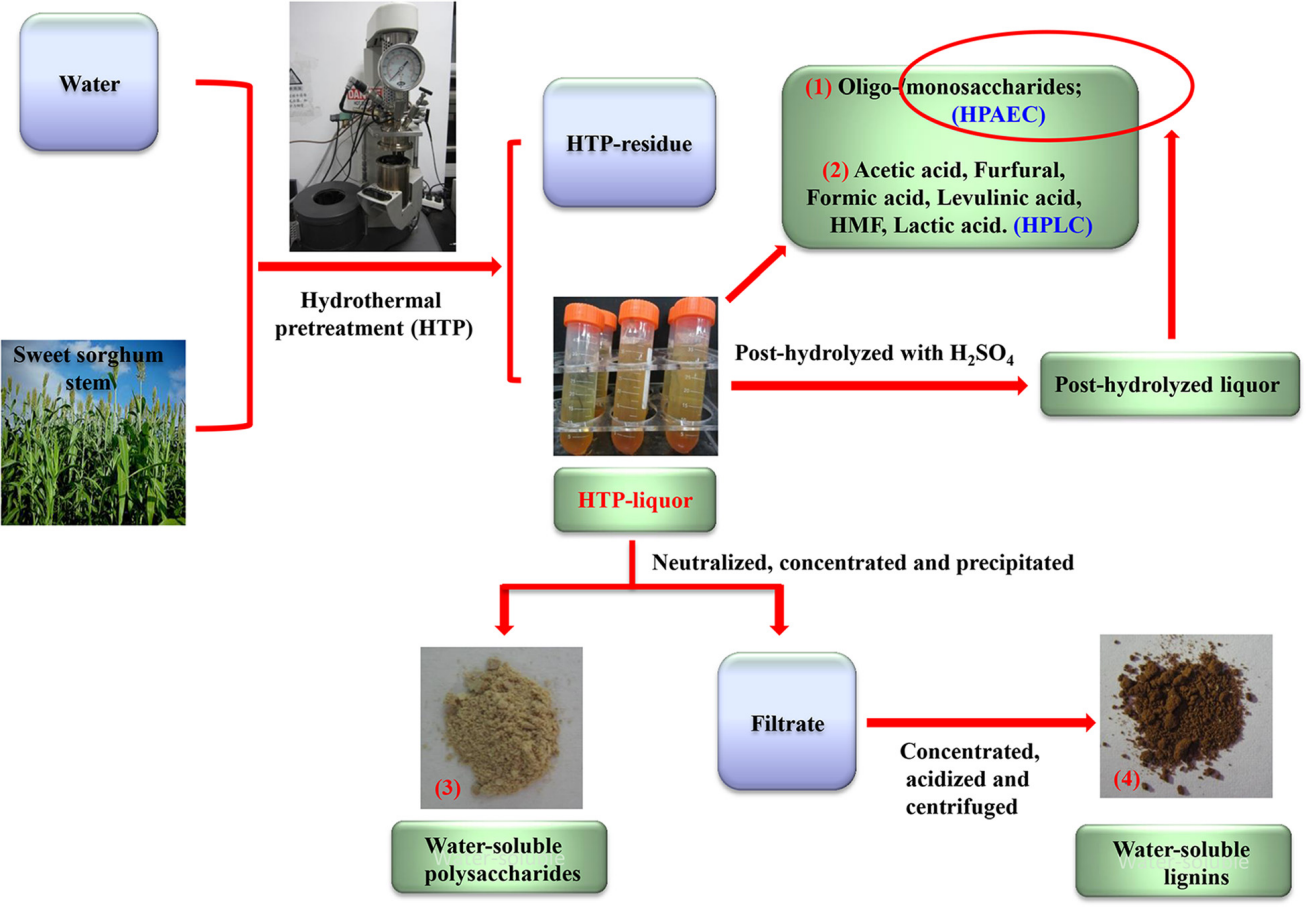
**Schematic illustration of the experimental procedure.**

In this study, the sweet sorghum stems (SSS) were subjected to different HTP conditions (110°C to 230°C, 0.5 to 2.0 h). For the substrates obtained after the HTP process, the enzymatic hydrolysis efficiency and structural properties have been thoroughly investigated in a recent publication [13]. This work mainly focuses on chemical characteristics of the degraded products in the liquor phase produced during the HTP process. Among them, the XOS, monosaccharides, and degree of polymerization of XOS were analyzed by high-performance anion exchange chromatography (HPAEC). Structural features of the XOS were identified by two-dimensional heteronuclear single quantum coherence (2D-HSQC). The chemical features (composition, molecular weights, and structural features) of the water-soluble polysaccharides and lignins (WSPs and WSLs) obtained from the liquor phase during the HTP process were systematically evaluated by HPAEC, gel permeation chromatography (GPC), and 2D-HSQC NMR, which will contribute to the potential applications in bioethanol industry under the biorefinery scenario. The schematic diagram of the experimental procedure is illustrated in Figure 1.

## Results and discussion

### The liquor pH

The pH of the initial liquor before heating was 6.80, and it decreased to 3.13 with the increasing HTP temperature and time (Table 1). This is expected that the hydronium ions released by the water during the HTP cause depolymerization of hemicelluloses by selective hydrolysis of glycosidic linkages, liberating *O*-acetyl group and other acid moieties to form various acids (such as acetic and uronic acids). The release of these acids is thought to catalyze the hydrolysis of hemicelluloses and oligosaccharides, resulting in more hemicelluloses that were degraded and solubilized in the liquor as the pretreatment temperature and time increased [14]. This is also the main reason for decreasing solid yields from 92.4% to 51.5% as the pretreatment temperature and time increased. Similarly, the strong positive correlations between the pH of the liquor and the solid yield have been also observed in a previous study [15].

**Table 1:** **The liquor pH and the degraded products of polysaccharides from sweet sorghum stem during the hydrothermal pretreatment process**

**Temperature (°C)-time (h)**	**Degraded products (g/L)**						**pH**
	**Formic acid**	**Acetic acid**	**Levulinic acid**	**Lactic acid**	**HMF**	**Furfural**	
110-1.0	ND	ND	ND	ND	ND	ND	5.18
130-1.0	ND	ND	ND	ND	ND	ND	4.50
150-1.0	1.10	0.38	0.52	ND	0.06	ND	3.78
170-0.5	0.85	0.48	0.74	0.28	0.12	0.18	3.65
170-1.0	1.96	0.63	1.39	0.37	0.21	0.85	3.48
170-2.0	2.26	1.01	2.26	0.73	0.32	2.11	3.30
190-0.5	2.56	1.26	2.43	1.65	0.38	3.32	3.22
210-0.5	3.16	1.64	2.95	2.20	1.23	3.50	3.18
230-0.5	3.76	4.03	3.65	2.41	2.27	4.53	3.13

ND, not detectable. Data represented are the averages of the results obtained from the duplicated experiments.

### Xylo-oligosaccharides and monosaccharides

**Figure 2:**
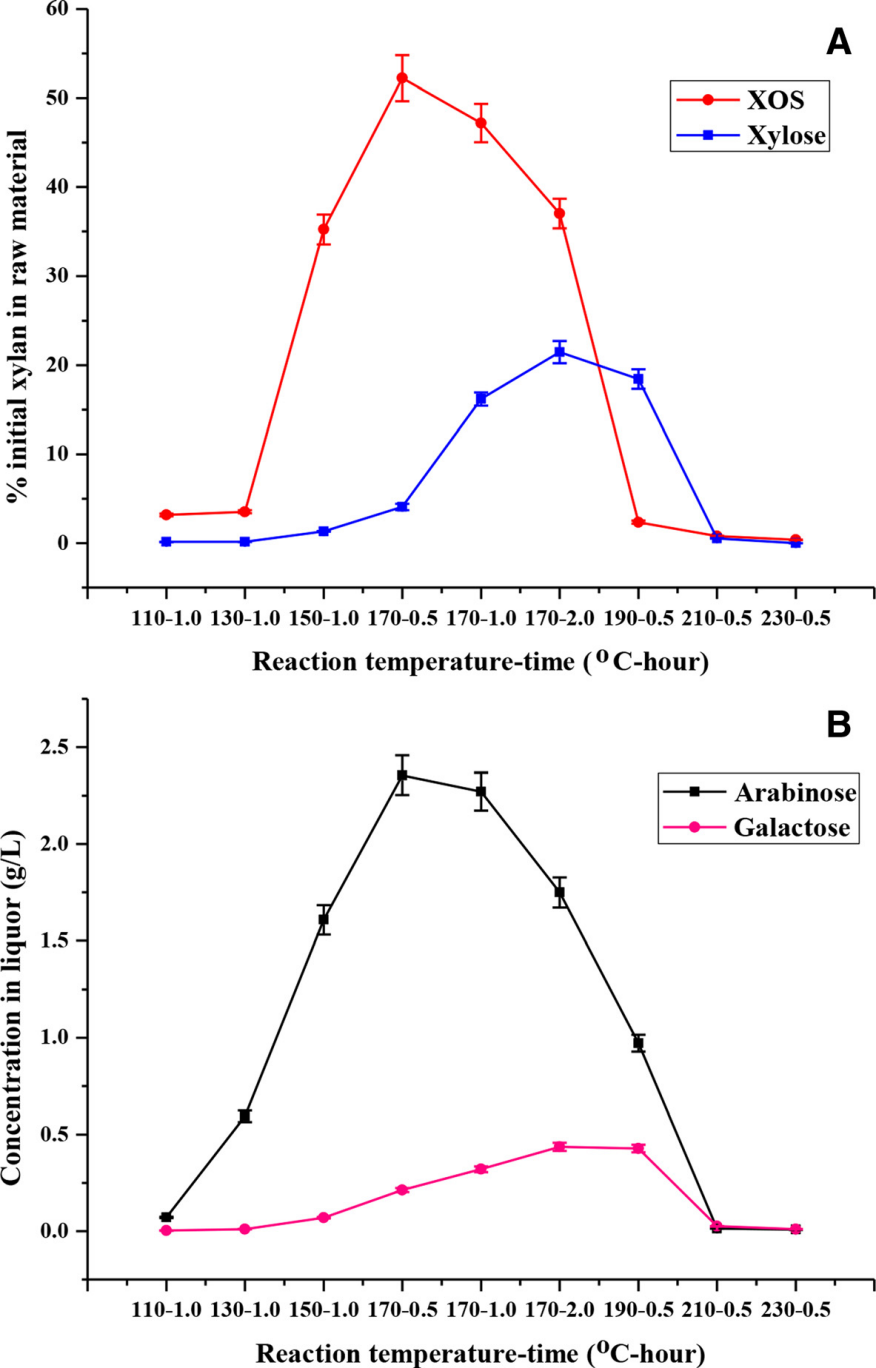
**Experimental values for (A) XOS and xylose, (B) arabinose, and galactose obtained during the hydrothermal pretreatment at 110°C to 230°C.** The yields of XOS and xylose are expressed as the percentage of the xylan in raw materials. The concentrations of arabinose and galactose are expressed as the concentration in the liquor phase. The error bars are standard deviations from experiments performed in duplicate. XOS, xylo-oligosaccharides.

Hemicelluloses (xylan-type) in lignocelluloses are hydrolyzed to xylose or xylo-oligomers during the HTP [6,16,17]. In general, the degree of xylan hydrolysis increases as the HTP severity increases [14]. In this study, the hydrolysis products in the liquor were mainly XOS and monosaccharides. Figure 2 shows the yields of XOS and monosaccharides as the pretreatment temperature and time increased during the HTP. As can be seen (Figure 2A), the yields of XOS (based on the initial xylan in raw material) steadily increased from 3.18% to 47.20% with an increase in the pretreatment temperature from 110°C to 170°C for a constant time (1.0 h). The reason for this is that more xylan was degraded and solubilized in the liquor at the elevated temperatures. Specially, the maximum yield of XOS (52.25%) was achieved in a relatively short period (0.5 h) at 170°C. When the pretreatment time was further prolonged to 1.0 and 2.0 h at 170°C, the yield of XOS decreased constantly with the prolongation of reaction time. The fact suggested that the prolongation of reaction time was adverse to the production of XOS at 170°C for 0.5 h. As expected, when the pretreatment temperature was higher than 170°C at a constant short time (0.5 h), the yields of XOS constantly decreased from 52.25% to 0.37% (in the experiment performed at 230°C). This was attributed to the fact that almost all the xylan was degraded into the xylose and other small molecules, resulting in only a small amount of XOS detected in the liquor phase under this condition [18]. Taken together, the optimal yield of XOS was achieved in the case of the material pretreated at 170°C for 0.5 h. Hence, this condition was considered as a promising pathway to obtain high-yield of XOS from SSS by the HTP process.

Figure 2A also illustrates that the yield of xylose (based on the initial xylan in raw material) was obtained at various pretreatment conditions. When the pretreatment time was 1.0 h, a significant enhancement of xylose concentration was observed as the pretreatment temperature increased from 110°C to 170°C, and a higher pretreatment temperature and longer pretreating period resulted in a rapid raise of xylose. The maximum yield of xylose was achieved (21.47% of the initial xylan) at 170°C for 2.0 h. The concentration variations of arabinose and galactose at various pretreatment conditions are shown in Figure 2B. The change of galactose concentration was quite similar to that of xylose, whose maximum concentration was also obtained (0.44 g/L) at 170°C for 2.0 h. The highest arabinose concentration was observed (2.36 g/L) at 170°C for 0.5 h. Under the optimal pretreatment condition for XOS (170°C for 0.5 h), the concentrations of arabinose and galactose were 2.36 and 0.21 g/L, respectively. Moreover, low concentrations of glucose were detected in the liquors pretreated at harsh conditions (that is, 0.59 g/L at 210°C for 0.5 h and 0.48 g/L at 230°C for 0.5 h, respectively), indicating that the part of non-crystalline cellulose may be hydrolyzed under the conditions given.

### Degree of polymerization and HSQC analysis of xylo-oligosaccharides

**Figure 3:**
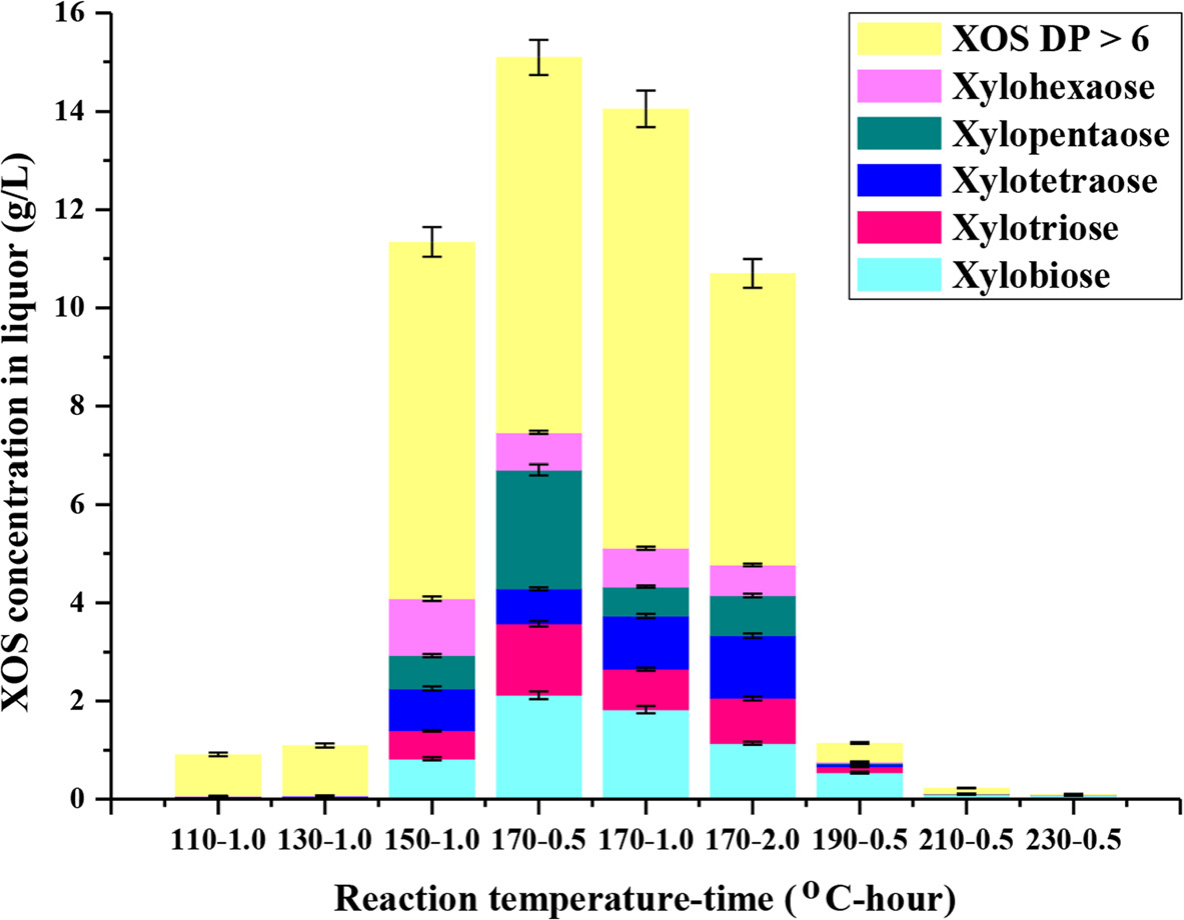
**The concentration of xylo-oligosaccharides with different degrees of polymerization.** The error bars are standard deviations from experiments performed in duplicate.

A systematic study was proposed for the production, purification, and applications of XOS as food additives and nutraceuticals. Considered as food ingredients, XOS have favorable modulation on the intestinal function [19]. In the present study, the degree of polymerization (DP) of XOS obtained during the HTP from SSS was determined. Figure 3 presents the concentration and DP distribution of XOS. The concentrations of xylobiose, xylotriose, xylotetraose, xylopentaose, and xylohexaose were determined by HPAEC. The total concentration of XOS was determined by an increasing concentration of monosaccharides after post-hydrolysis.

The DP of XOS illustrated that it was influenced by the pretreatment temperature and time. As the pretreatment temperature increased (110°C to 170°C, 1.0 h), more low-DP XOS (DP 2 to 6) were obtained. However, when the pretreatment temperature further increased from 170°C to 230°C, the yields of low-DP XOS (DP 2 to 6) rapidly declined with the pretreatment severity. The maximum yield of XOS was achieved with 14.05% xylobiose (2.12 g/L), 9.61% xylotriose (1.45 g/L), 4.71% xylotetraose (0.71 g/L), 16.10% xylopentaose (2.43 g/L), 5.04% xylohexaose (0.76 g/L), and 50.49% high-DP XOS (DP > 6) (7.63 g/L) at 170°C for 0.5 h. However, when the reaction time was prolonged to 1.0 h at 170°C, the yield of low-DP XOS (DP 2 to 6) increased but the high-DP XOS (DP > 6) decreased. After further prolonging the reaction time to 2.0 h, the low and high-DP XOS simultaneously reduced. These phenomena were probably due to the XOS that was further degraded into other small molecules (such as furfural) under the harsh conditions, as revealed by the subsequent degraded products analysis in the liquors produced during the HTP from SSS.

**Figure 4:**
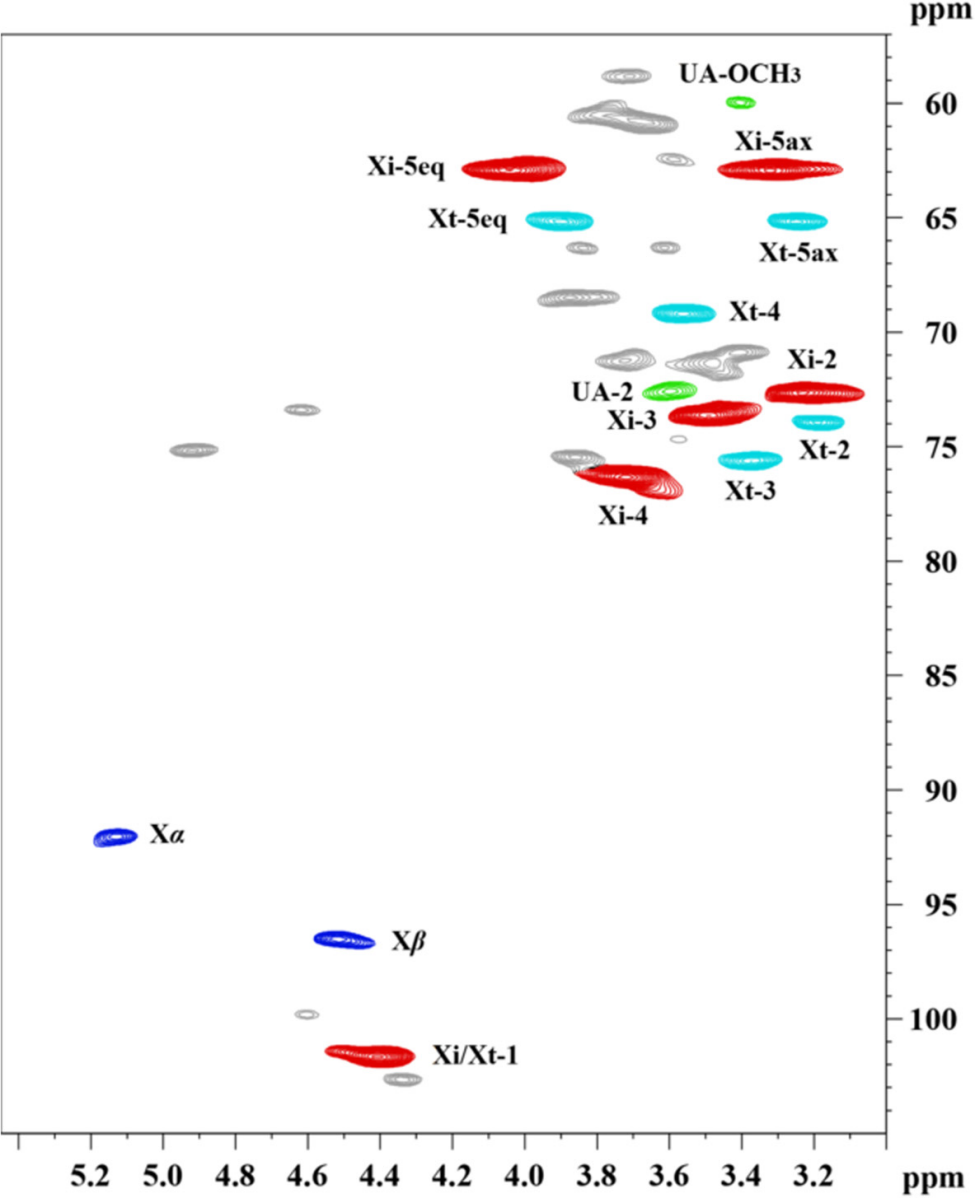
**HSQC analysis of xylo-oligosaccharides obtained during the hydrothermal pretreatment at 170°C for 0.5 h.** HSQC, heteronuclear single quantum coherence; Xα and Xβ, xylopyranosyl reducing ends; Xi and Xt, internal and nonreducing ends; UA, 4-*O*-methyglucuronic acid.

A 2D-HSQC spectrum (Figure 4) was used to identify the various structural features of XOS obtained under the pretreated at 170°C for 0.5 h. The signals of the XOS were interpreted as follows: (a) reducing end xylopyranosyl (Xylp) residues of *α* and *β* conformations (*δ*_C_/*δ*_H_ 92.0/5.13 and 96.5/4.52); (b) internal Xylp residues (1 → 4)-*β*-D-Xylp, that is, *δ*_C_/*δ*_H_ 101.6/4.40 (C_1_-H_1_), 72.6/3.23 (C_2_-H_2_), 73.6/3.49 (C_3_-H_3_), 76.3/3.72 (C_4_-H_4_), 62.9/4.04 (C_5eq_-H_5eq_), and 62.9/3.32 (C_5ax_-H_5ax_); (c) terminal (non-reducing end) Xylp residues (*β*-D-Xylp-(1-, that is, *δ*_C_/*δ*_H_ 75.6/3.37 and 69.2/3.56); and (d) a small amount of Xylp units attached with 4-*O*-Me-*α*-D-GlcpA units (*δ*_C_/*δ*_H_ 72.6/3.60 and 59.9/3.40). Many groups exhibited unequal intensities, indicating that the XOS contained a mixture of several molecules. The XOS showed a low degree of branching according to the weak signals of 4-*O*-Me-*α*-D-GlcpA residues. Note that the structural features of XOS are dependent not only on the origin of the xylan-rich hemicelluloses but also on the process [20]. For example, XOS prepared from xylan-rich hemicelluloses, isolated using aqueous potassium hydroxide from delignified sugarcane bagasse, by hydrolysis with crude xylanase secreted by *Pichia stipitis* contained Araf and 4-*O*-Me-*α*-D-GlcpA residues [21].

### Degraded products of polysaccharides

The main degraded products of polysaccharides from lignocelluloses during the HTP were acetic acid, furfural, hydroxymethylfurfural (HMF), and formic acid, which were described in detail in a recent publication [18]. As can be seen, hemicelluloses are partially acetylated and the acetyl ester bonds are hydrolyzed to produce acetic acid under the HTP process. Furfural is a pentose dehydration product and can be further degraded through hydrolytic fission of the aldehyde group to formic acid under the acidic hydrothermal condition [22]. HMF is a dehydration product of hexose sugars (glucose, galactose, and mannose) and can be further transformed into levulinic and formic acids under the acidic condition [23]. These degraded products are undesirable, since they act as inhibitors of the fermentation process [24].

In this study, acetic acid, furfural, HMF, formic acid, levulinic acid, and lactic acid were found to be the main degraded products detected in the liquor fractions. The concentrations of the degraded products of polysaccharides in the liquor phase were listed in Table 1. The concentrations of acetic acid (4.03 g/L) and furfural (4.53 g/L) were significantly higher than other degraded products (<3.76 g/L) at the severest pretreatment condition. It was interesting to note that the release of all degraded products was closely related to the pretreatment temperature and time, that is, the concentrations of all degraded products increased with increasing temperature at a constant time or increasing time at a constant temperature. As expected, the maximum concentrations of all degraded products were observed in the case of the material pretreated at 230°C for 0.5 h, corresponding to the severest experimental condition in this work. It appears that the HTP condition under the higher HTP temperature at a short reaction time (that is, 230°C for 0.5 h) favors the dehydration of xylose to furfural as compared to that obtained at other experiment conditions. The concentration of HMF remained low throughout among the whole range of pretreatment severities due to limited cellulose solubilization and consequent glucose formation. The concentration of levulinic acid was much lower than that of formic acid, suggesting that most of formic acid was produced from furfural and not from HMF degradation, since the latter would lead to the production of equimolar amounts of levulinic and formic acids [18].

### Water-soluble polysaccharides

The composition of the WSPs obtained from the HTP-liquor phase is presented in Table 2. These WSPs contained most xylose and glucose together with small amount of arabinose, galactose, rhamnose, mannose, and uronic acids (glucuronic and galacturonic acids). Under the HTP temperature lower than 210°C, the content of xylose was apparently higher than glucose in the WSPs. In contrast, as the HTP temperature over 190°C, the content of glucose predominates over xylose in the WSPs. The high content of glucose in the two WSPs (WSP_210(0.5)_ and WSP_230(0.5)_) was probably related to the hydrolysis of amorphous cellulose under the harsh conditions. The amorphous cellulose may be hydrolyzed to the cellulosic fragment, resulting in part of cellulose that was co-precipitated in the WSPs. Table 3 exhibits weight average (*M*_*w*_) and number average (*M*_*n*_) molecular weights along with polydispersity indexes (PDIs, *M*_*w*_/*M*_*n*_) of the WSPs. The *M*_*w*_ and *M*_*n*_ of the WSPs gradually reduced as the pretreatment temperature and time increased, implying that the C-O bonds of the WSP was gradually cleaved, especially at the harsh conditions (≥170°C). In addition, these WSPs with lower PDIs (1.10 to 2.01) suggested that the HTP process yielded more homogeneous polysaccharide polymers.

**Table 2:** **Relative content of sugars and uronic acids of the water-soluble polysaccharides isolated from the hydrothermally pretreated liquor phase under the various processing conditions**

**Temperature (°C)-time (h)**	**Neutral sugars and uronic acids**						
	**Rhamnose**	**Arabinose**	**Galactose**	**Glucose**	**Xylose**	**Mannose**	**Uronic acid**
110-1.0	1.76	21.34	10.13	27.48	33.69	0.94	4.66
130-1.0	0.95	21.02	10.24	26.63	37.86	0.29	3.01
150-1.0	ND	6.27	6.07	21.04	65.56	ND	1.06
170-0.5	ND	3.42	4.17	14.59	77.28	ND	0.54
170-1.0	ND	6.72	5.49	21.02	66.77	ND	ND
170-2.0	ND	6.50	4.17	20.23	69.10	ND	ND
190-0.5	ND	5.76	3.02	18.64	72.58	ND	ND
210-0.5	ND	ND	2.63	81.16	16.21	ND	ND
230-0.5	ND	ND	ND	82.68	17.32	ND	ND

ND, not detectable. Data represented are the averages of the results obtained from the duplicated experiments.

**Table 3:** **Weight-average (** ***Μ***_**w**_ **) and number-average (** ***Μ***_**n**_ **) molecular weights and polydispersits (** ***Μ***_**w**_ **/** ***Μ***_**n**_ **) of the water-soluble polysaccharides isolated from the hydrothermally pretreated liquor phase under the various processing conditions**

	**Water-soluble polysaccharides (temperature (°C)-time (h))**								
	**110-1.0**	**130-1.0**	**150-1.0**	**170-0.5**	**170-1.0**	**170-2.0**	**190-0.5**	**210-0.5**	**230-0.5**
*M*_*w*_	31,220	25,920	16,830	14,320	7,110	6,710	7,080	6,190	4,780
*M*_*n*_	15,550	14,160	8,520	7,670	6,240	5,930	6,150	5,620	4,020
*M*_*w*_/*M*_*n*_	2.01	1.83	1.98	1.88	1.14	1.13	1.15	1.10	1.19

Data represented are the averages of the results obtained from the duplicated experiments.

**Figure 5:**
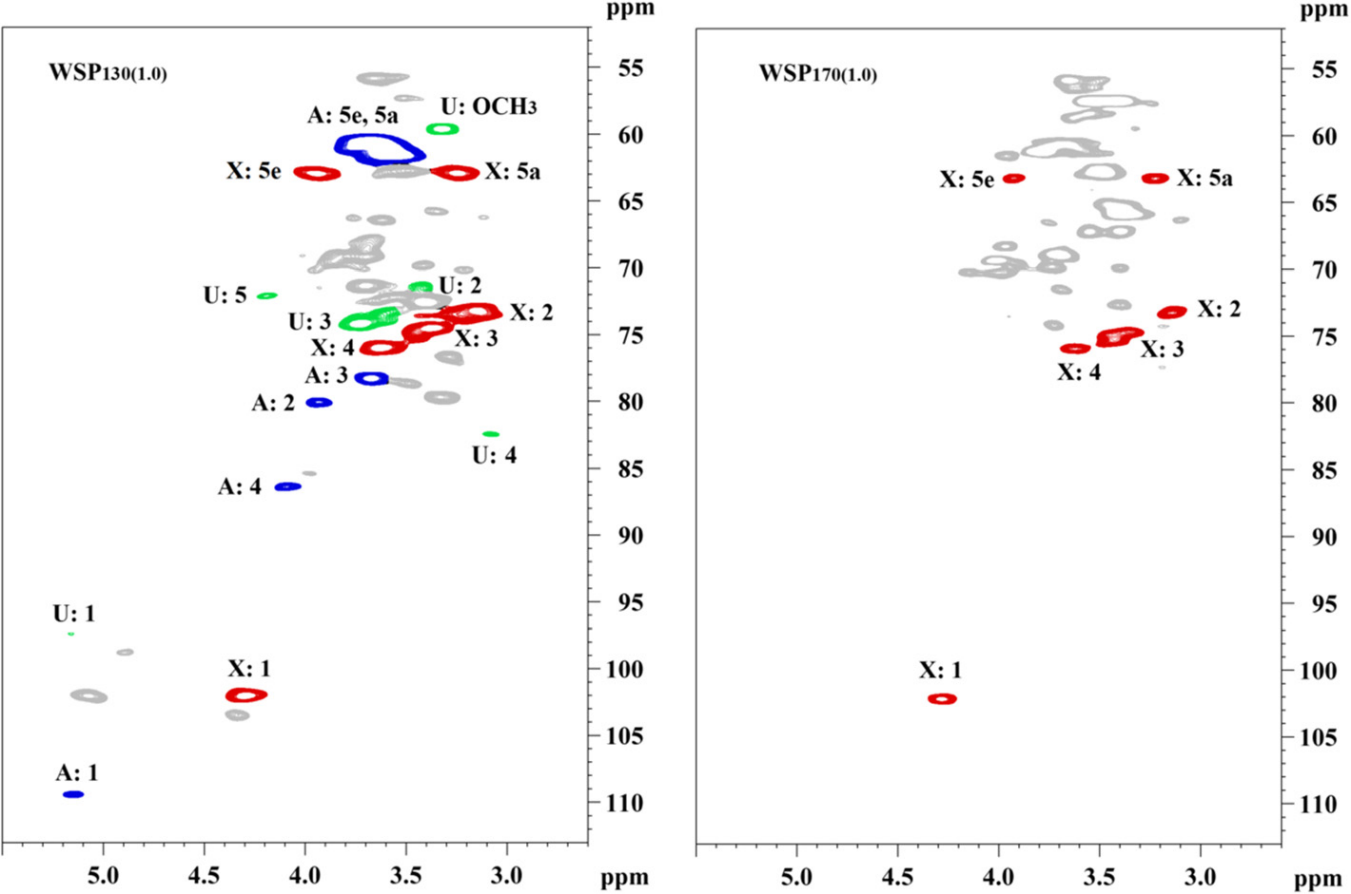
**HSQC spectra of water-soluble polysaccharides (WSP**_**130(1.0)**_ **and WSP**_**170(1.0)**_ **).** HSQC, heteronuclear single quantum coherence; WSP, water-soluble polysaccharide; X, xylopyranosyl units; A, arabinofuranosyl units; U, 4-*O*-methyglucuronic acid.

Structural features of the WSPs (WSP_130(1.0)_ and WSP_170(1.0)_) were determined by 2D-HSQC NMR technique (Figure 5). Comparison of HSQC spectra of the WSPs obtained from the liquor phase can help to better understand the structural variation of the WSPs during the HTP process. For WSP_130(1.0)_, cross signals of (1 → 4)-linked *β*-D-Xyl*p* units at *δ*_C_/*δ*_H_ 102.0/4.31 (C_1_-H_1_), 73.2/3.15 (C_2_-H_2_), 74.5/3.38 (C_3_-H_3_), 76.0/3.63 (C_4_-H_4_), 62.9/3.94 (C_5eq_-H_5eq_), and 63.3/3.25 (C_5ax_-H_5ax)_ were clearly observed. The signals of *α*-L-arabinofuranosyl units were found at *δ*_C_/*δ*_H_ 109.4/5.15, 80.1/3.93, 78.3/3.67, 86.3/4.09, and 61.2/3.65, which are characteristic of C_1_-H_1_, C_2_-H_2_, C_3_-H_3_, C_4_-H_4_, and C_5_-H_5_, respectively. Some weak signals at *δ*_C_/*δ*_H_ 97.4/5.16, 71.5/3.42, 74.0/3.73, 82.4/3.08, 72.1/4.19, and 59.6/3.33 are assigned to C_1_-H_1_, C_2_-H_2_, C_3_-H_3_, C_4_-H_4_, C_5_-H_5_, and -OCH_3_ of 4-*O*-Me-*α*-D-Glc*p*A units, respectively [25,26]. As the HTP temperature was further increased to 170°C and kept for 1.0 h, only the cross signals of *β*-D-Xyl*p* units were observed, implying that the xylan-rich polymers (WSP_170(1.0)_) were collected. This is because that the side chains of the WSP macromolecules were significantly cleaved under this condition. These results suggested that the degradation degree of xylans increased with the HTP temperature increase, as revealed by the aforementioned molecular weight analysis.

### Water-soluble lignins

**Figure 6:**
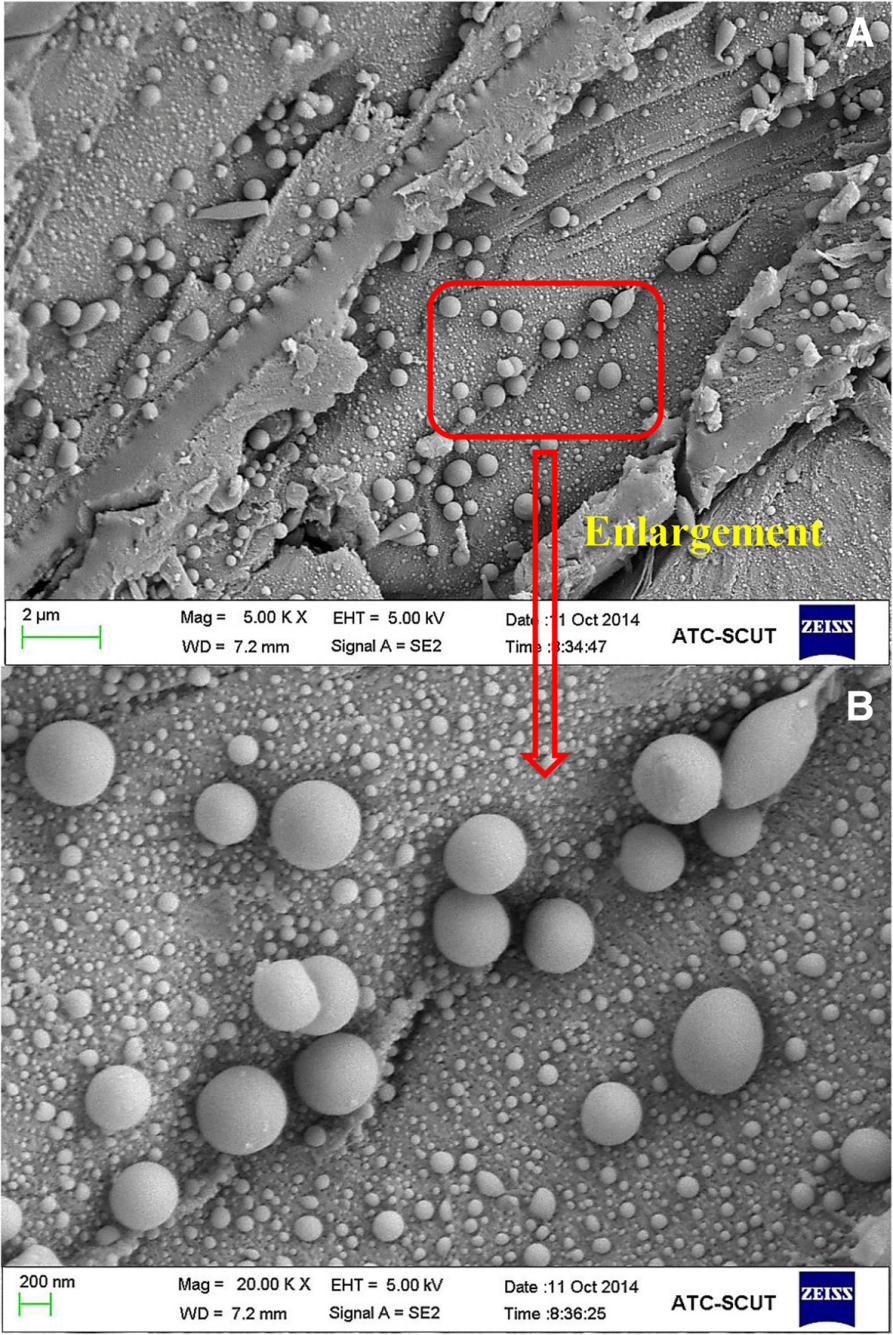
**SEM images of the hydrothermally pretreated substrate at 210°C for 0.5 h. (A)** Magnification × 5,000. **(B)** Magnification × 20,000. SEM, scanning electron microscopy.

Lignin is considered the most persistent component of the plant cell wall biopolymers [27]. It is found primarily in the secondary cell wall and plays a major role in pathogen resistance, water regulation, and conferring strength for the integrity of the cell wall structure. The effects of lignin on biomass enzymatic digestibility have received extensive attention [10,28]. It is generally recognized that the existence of lignins in lignocelluloses restricts enzymatic saccharification by physically impeding the accessibility of cellulase to cellulose [10]. HTP causes fragmentation of lignins, which results in the redistribution of lignins and its structural changes depending on the HTP severity [29]. It has been reported that a decrease in molecular weight of native lignins would facilitate its dissolution and/or migration to the surface in the reaction media during the HTP [10]. The spherical droplets found in the pretreated hardwood are thought to be mainly composed of lignins based on previous reports [30,31]. In addition, pseudo-lignin derived from the dehydrated carbohydrates during severe HTP was also suggested to be responsible for the formation of droplets [32,33]. In this study, scanning electron microscopy (SEM) images demonstrated that the harsh HTP process (210°C for 0.5 h) prompted migration or formation of lignins or pseudo-lignin (Figure 6A). Figure 6B shows a partial enlargement, revealing in more spherical droplets with different sizes. To sum up, SEM images suggested that the lignin or pseudo-lignin during the harsh HTP has inhomogeneous morphological features. To prove the fragmentation of lignins during the HTP, the molecular weights of the WSLs (WSL_110(1.0)_, WSL_130(1.0)_, WSL_150(1.0)_, and WSL_170(1.0)_) were determined by GPC analysis (Table 4). It can be seen that an elevated HTP temperature caused the tremendous decrease of molecular weight in the WSLs, especially at higher temperature condition. Several researchers have investigated the evaluation of structural changes of lignins during the HTP process, and the results suggest that there is no significant decrease in the molecular weight of lignins. This was attributed to the partial protection of lignins by polysaccharides in the compact biomass matrix, which may facilitate the simultaneous re-condensation process occurring along with depolymerization [34]. Moreover, the polydispersity indices of the WSLs were slightly decreased (1.48 to 1.13) with an increasing pretreatment temperature from 110°C to 170°C for 1.0 h, suggesting that more homogeneous lignin fragments were obtained under the harsh condition (170°C for 1.0 h).

**Table 4:** **Weight-average (** ***Μ***_**w**_ **) and number-average (** ***Μ***_**n**_ **) molecular weights and polydispersity indices (** ***Μ***_**w**_ **/** ***Μ***_**n**_ **) of the water-soluble lignins isolated from the hydrothermally pretreated liquor phase under the various processing conditions**

	**Water-soluble lignins (temperature (°C)-time (h))**			
	**110-1.0**	**130-1.0**	**150-1.0**	**170-1.0**
*M*_w_	1,610	1,590	1,050	950
*M*_n_	1,090	1,220	900	840
*M*_w_/*M*_n_	1.48	1.30	1.17	1.13

Data represented are the averages of the results obtained from the duplicated experiments.

**Figure 7:**
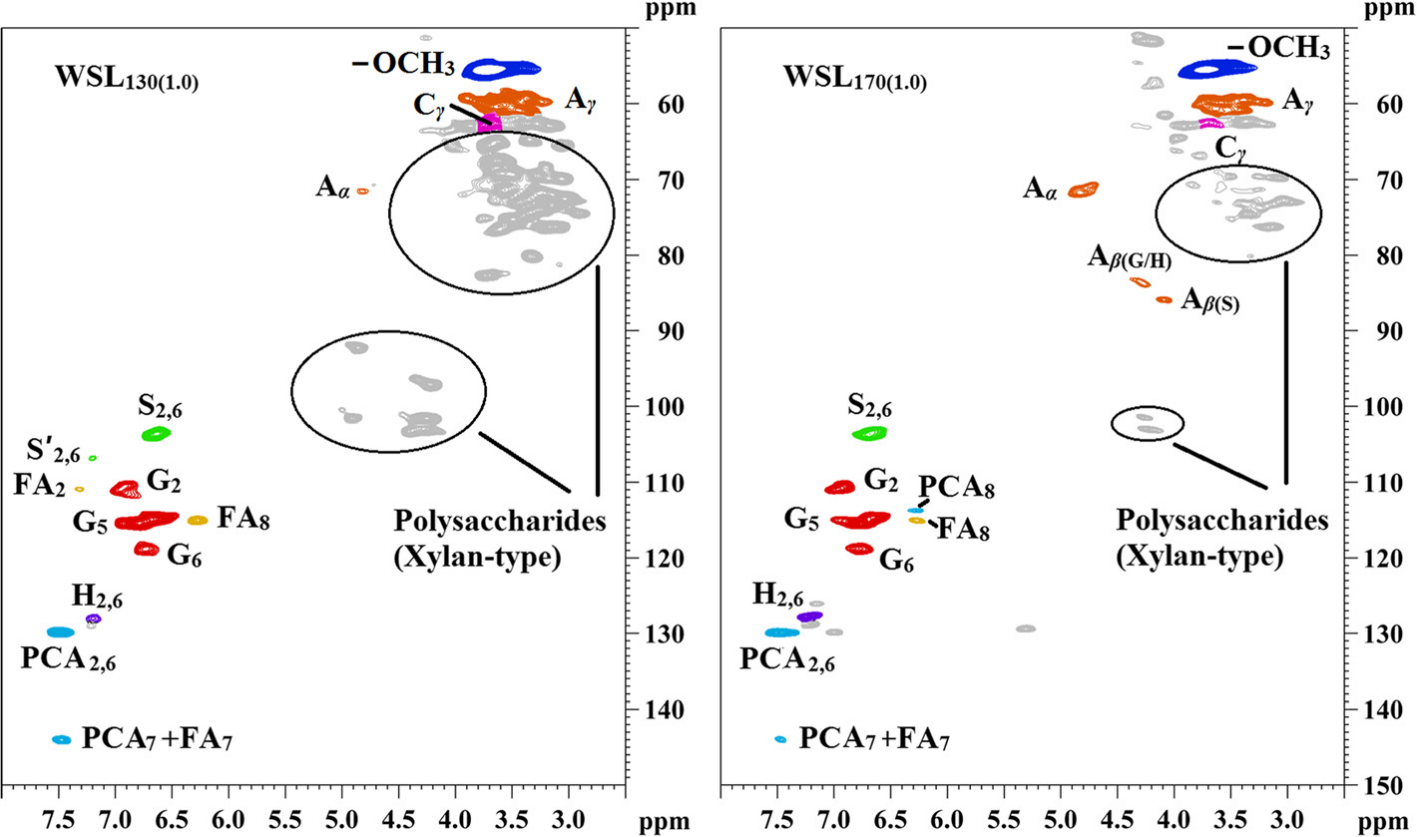
**HSQC spectra of water-soluble lignins (WSL**_**130(1.0)**_ **and WSL**_**170(1.0)**_ **).** HSQC, heteronuclear single quantum coherence; WSL, water-soluble lignin; A, *β*-aryl-ether units (*β*-*O*-4); C, phenylcoumaran substructures (*β*-5); PCA, free *p*-coumaric acid; FA, ferulate; H, *p*-hydroxyphenyl units; G, guaiacyl units; S, syringyl units; S’, oxidized syringyl units bearing a carbonyl at C_*α*_.

To further reveal structural differences of the WSLs (WSL_130(1.0)_ and WSL_170(1.0)_) obtained from the liquor phase, the more detailed structural information was investigated by 2D-HSQC NMR spectra (Figure 7) [35-37]. The inter-unit linkages in the WSLs, *β*-ether (A, *β*-*O*-4) and phenylcoumaran (C, *β*-5) were identified due to the presence of cross-peaks at *δ*_C_/*δ*_H_ 71.5/4.82 (A_*α*_), 60.2/3.60 (A_*γ*_), and 62.5/3.67 (C_*γ*_), respectively. Moreover, the cross-peaks of methoxy groups in the WSLs (−OCH_3_, *δ*_C_/*δ*_H_ 55.5/3.72) were clearly observed. The aromatic lignin units syringyl (S), guaiacyl (G) and *p*-hydroxyphenyl (H) units showed prominent correlations at *δ*_C_/*δ*_H_ 103.6/6.63 (S_2,6_), 110.7/6.93 (G_2_), 115.4/6.78 (G_5_), 118.8/6.73 (G_6_), 128.0/7.19 (H_2,6_), respectively. Minor amounts of oxidized S units (S’) were detected due to the presence of a correlation at *δ*_C_/*δ*_H_ 106.8/7.20 (S’_2,6_). In addition, the WSLs obtained appeared to contain small amounts of *p*-hydroxycinnamates (*p*-coumaric and ferulic acids). The *p*-coumaric acid (PCA) was characterized by some relatively intense correlations at *δ*_C_/*δ*_H_ 129.8/7.49 (PCA_2,6_), 115.0/6.27 (PCA_8_), and 144.0/7.47 (PCA_7_), while ferulic acid (FA) was found at *δ*_C_/*δ*_H_ 110.9/7.31 (FA_2_), 115.0/6.27 (FA_8_), and 144.0/7.47 (FA_7_). As can be seen, when the pretreatment temperature reached 170°C, some expected changes appeared in the HSQC spectrum of WSL_170(1.0)_ as compared to that of WSL_130(1.0)_. For example, the correlated signals (*δ*_C_/*δ*_H_ 71.5/4.82, 60.2/3.60, 83.6/4.29, and 85.2/4.08) of *β*-*O*-4 were dramatically increased in WSL_170(1.0)_. Meanwhile, the signal strength of polysaccharides reduced in WSL_170(1.0)_. This was mainly attributed to the degradation of polysaccharides, especially under the higher temperatures. The facts showed that more pure lignins were achieved under this condition. Besides, no signal of S’ was detected in WSL_170(1.0)_, implying that the oxidized S units occurred at a relatively lower temperature for a shorter time of pretreatment. The correlated signals of PCA were increased while the signals of FA were decreased as the pretreatment temperature increased from 110°C to 170°C for 1.0 h.

### Process mass balance

**Figure 8:**
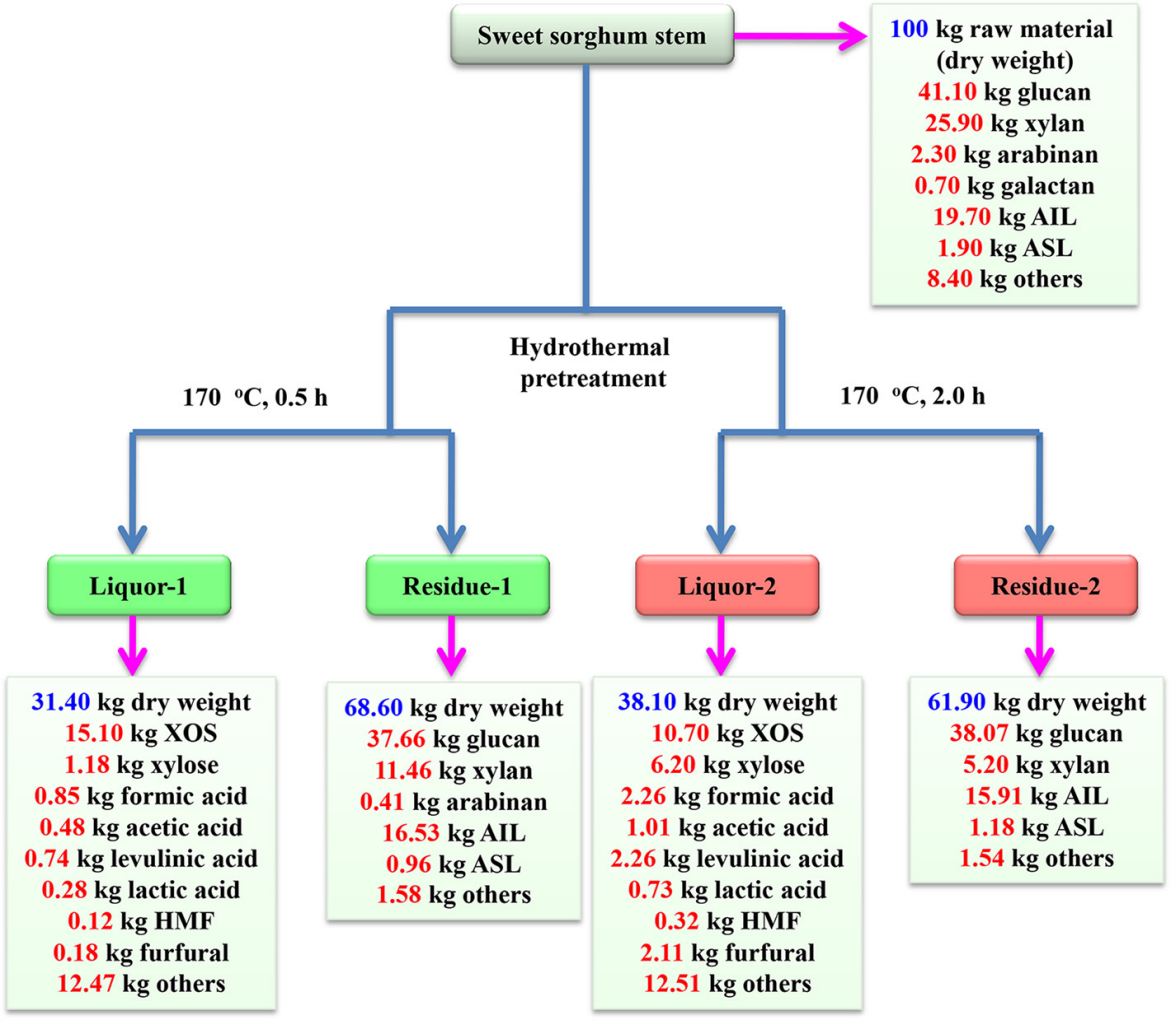
**Mass balance during the pretreatment at 170°C for 0.5 and 2.0 h.**

In this study, the maximum yield of XOS was achieved at 170°C for 0.5 h with a relatively low level of xylose and other by-products. Prolonged HTP time and higher temperature reduced the yield of XOS and enhanced the concentrations of monosaccharides and by-products. Therefore, a process mass balance of the HTP process was developed for the two HTP conditions (0.5 and 2.0 h at 170°C) (Figure 8). Process yield was normalized to a common basis of 100 kg of dried SSS as the starting material [38]. It should be noted that, after the HTP, the liquor and solid were separated by filtration. The yields of the HTP residues decreased from 68.60 to 61.90 kg with an increasing HTP time from 0.5 to 2.0 h at 170°C [39]. It was found that 15.10 and 10.70 kg XOS could be obtained when the HTP processes were performed at 170°C for 0.5 and 2.0 h, respectively (Figure 8). In addition, other more degraded products appeared in the liquor fractions for the prolonged HTP period, which was attributed to the significant degradation of hemicelluloses under the harsh HTP conditions.

## Conclusions

The present study demonstrated that the hydrothermally pretreated conditions had significant influences on the compositions and their chemical structures of the degraded products. It was found that the maximum yield of XOS of 52.25% was achieved at 170°C for 0.5 h with a relatively low level of xylose and other degraded products. Higher temperature and/or longer reaction time reduced the yield of XOS and enhanced concentrations of monosaccharides (arabinose and galactose). In addition, as the pretreatment temperature and time increased, the concentrations of the degraded products (such as acetic acid and furfural) were dramatically increased. Furthermore, the 2D-HSQC spectrum of the XOS suggested that the XOS obtained mainly contained xylopyranosyl residues together with small amounts of 4-*O*-Me-*α*-D-GlcpA units. Molecular weights of the water-soluble polysaccharides and lignins implied that the degradation of the polysaccharides and lignins occurred at the harsh conditions. Interestingly, 2D-HSQC spectra suggested that the water-soluble polysaccharides (rich in xylan) and water-soluble lignins (rich in *β*-*O*-4 linkages) were obtained at 170°C for 1.0 h. In short, systematic evaluation of the degraded products during the hydrothermal pretreatment of SSS will be beneficial to value-added applications of multiple chemicals in a biorefinery process for bioethanol industry.

## Methods

### Raw materials

SSS was obtained from the experimental farm of the North-Western University of Agricultural and Forest Sciences and Technology (Yangling, Shaanxi, China). As determined by the National Renewable Energy Laboratory (NREL) using a two-step acid hydrolysis procedure, the extractive-free SSS was found to contain 41.1% glucan, 25.9% xylan, 2.3% arabinan, 0.7% galactan, and 21.4% lignin (19.5% Klason lignin and 1.9% acid-soluble lignin) [40]. More details of the materials used in this study were given in a recent literature [26]. XOS (DP 2 to 6) standards were obtained from Megazyme (Megazyme International Ireland Ltd., Wicklow, Ireland). All chemicals were analytical grade and purchased from Sigma-Aldrich (Beijing, China).

### Hydrothermal pretreatment

The HTP process was carried out in a 1,000-mL stainless steel autoclave (Parr Instrument Company, Moline, IL, USA) with a magnetic stirrer at a solid to liquor ratio of 1:10 (g/mL) by a PID controller (model 4848). The HTP experiments were divided into nine batches, in each batch, 15.0 g the extractive-free material was mixed with 150 mL deionized water. Then, the mixture was heated at 110°C, 130°C, or 150°C for 1.0 h, 170°C for different periods (0.5, 1.0, or 2.0 h), or 190°C, 210°C, or 230°C for 0.5 h. After the reaction, the reactor was cooled by cold water.

On the one hand, a liquor sample of 3 mL was post-hydrolyzed with 4% H_2_SO_4_ at 121°C for 1 h to determine the total concentration of XOS and their degree of substitution by acetyl group. The increased concentrations of monosaccharides and acetic acid after post-hydrolysis was regarded as the concentration of XOS and the amount of acetyl group attached on XOS, respectively [41,42]. According to this procedure, saccharides of DP 2 or higher were considered as XOS. This is different from the common definition of XOS which refers to a DP of 2 to 10. All the liquor samples were filtered through 0.22-μm filter before analysis. The concentration of XOS was expressed as monosaccharides equivalents.

On the other hand, the liquor (100 mL) was neutralized to pH 5.5 to 6.0 with 6 M NaOH and were further concentrated to 40 mL under vacuum. Subsequently, each concentrated solution was poured into 95% ethanol (120 mL) with vigorous stirring, and the WSP was obtained by filtering, washing with 70% aqueous ethanol. The supernatants were concentrated to 30 mL and poured in 150 mL acidic water (pH = 2.0, adjusted by HCl) to precipitate the WSL. The detailed procedures are previously described in a publication [43]. The WSP and WSL obtained were freeze-dried using a lyophilizer (Thermo Modulyo Freeze Dryer; Thermo Scientific, Waltham, MA, USA) at −50°C under vacuum for 48 h.

### Analysis procedures

HPAEC was used to quantify the monosaccharides and oligosaccharides of the liquor phase. An ICS-3000 HPAEC system with an AS50 autosampler, comprising a Carbopac PA-20 column (4 × 250 mm, Dionex, Sunnyvale, CA, USA) for monosaccharides and a Carbopac PA-100 column (4 × 250 mm, Dionex) for oligosaccharides were used. The quantitative analysis of the degraded products (acetic acid, furfural, formic acid, levulinic acid, HMF, lactic acid) was determined on a high-performance liquid chromatography (HPLC, Agilent 1200 series, Agilent Technologies Inc., Santa Clara, CA, USA) using the method described in a previous paper with minor modification [44]. The XOS powder obtained in the case of the material pretreated at 170°C for 0.5 h was characterized by HSQC NMR. HSQC experiment was conducted with 20 mg sample dissolved in 0.5 ml D_2_O. The number of collected complex points was 1,024 for the ^1^H-dimension with a relaxation of 1.5 s. The number of scans was 128, and 256 time increments were recorded in ^13^C-dimension. The 1*J*_*C*-*H*_ used was 146 Hz. Prior to Fourier transformation, the data matrixes were zero filled up to 1,024 points in the ^13^C-dimension. Substrate (hydrothermally substrate pretreated at 210°C for 0.5 h) was analyzed by SEM. SEM images were performed with a ZEISS EVO 18 (Carl Zeiss, Inc., Oberkochen, Germany). The accelerating voltage of the instrument was maintained at 5.00 and 20.00 kV.

The chemical compositions of the WSPs were analyzed by HPAEC. Each of the fractions (5 mg) was hydrolyzed with 10% H_2_SO_4_ at 105°C for 2.5 h. Then, the hydrolysate was filtrated, diluted 50-fold, and injected into a HPAEC system (Dionex ICS 3000) with an amperometric detector, an AS50 autosample, a CarbopacTM PA-20 column (4 × 250 mm, Dionex), and a guard PA-20 column (3 × 30 mm, Dionex). Neutral sugars and uronic acids were separated in a 5-mM NaOH isocratic (carbonate free and purged with nitrogen) for 20 min, followed by a 0 to 75 mM NaAc gradient in a 5-mM NaOH for 15 min. Then, the columns were washed with 200 mM NaOH for 10 min to remove the carbonate and followed by a 5-min elution with 5 mM NaOH to re-equilibrate the column before the next injection [45]. The *M*_w_ and *M*_n_ molecular weights of all the WSPs were determined by GPC, using a PL aquagel-OH 50 column (300 × 7.7 mm, Polymer Laboratories Ltd., Church Stretton, Shropshire, UK). The data were calibrated with PL pullulan polysaccharide standards. The detector used was a differential refractive index detector (RID). The eluent was 0.02 M NaCl in 0.005 M sodium phosphate buffer (pH 7.5), and the flow rate was 0.5 ml/min. Soluble-state NMR spectra were conducted on a Bruker AVIII 400 MHz spectrometer (Bruker AXS, Inc., Madison, WI, USA). HSQC NMR technique has also been applied to the structural determination of the WSPs based on the aforementioned conditions (XOS). The *M*_w_ and *M*_n_ of the WSLs were determined by GPC with an ultraviolet detector (UV) at 240 nm. The column used was a PL gel 10 mm mixed-B 7.5 mm i.d. column, which was calibrated with PL polystyrene standards. For 2D-HSQC spectra, the Bruker pulse program “hsqcetgpsi” was used and the parameters used is listed as below: the number of collected complex points was 1 K for the ^1^H dimension with d_1_ (2 s), number of scanning is 64, and 256 time increments were always recorded.

## Abbreviations

2D-HSQC: two-dimensional heteronuclear single quantum coherence
DP: degree of polymerization
FA: ferulic acid
G: guaiacyl
GPC: gel permeation chromatography
H: *p*-hydroxyphenyl
HMF: hydroxymethylfurfural
HPAEC: high-performance anion exchange chromatography
HPLC: high-performance liquid chromatography
HTP: hydrothermal pretreatment
*M*_*n*_: number-average
*M*_*w*_: weight-average
NREL: National Renewable Energy Laboratory’s
PCA: free *p*-coumaric acid
PDI: polydispersity indexes
RID: refractive index detector
S: syringyl
SEM: scanning electron microscopy
SSS: sweet sorghum stem
WSL: water-soluble lignins
WSP: water-soluble polysaccharide
XOS: xylo-oligosaccharides
